# House-Level Risk Factors for *Aedes aegypti* Infestation in the Urban Center of Castilla la Nueva, Meta State, Colombia

**DOI:** 10.1155/2021/8483236

**Published:** 2021-10-23

**Authors:** Adolfo Vásquez-Trujillo, Doris Cardona-Arango, Angela M. Segura-Cardona, Daniel C. Portela-Câmara, Nildimar Alves-Honório, Gabriel Parra-Henao

**Affiliations:** ^1^Escuela de Graduados, Universidad CES, Medellín, Colombia; ^2^Fundacao Oswaldo Cruz, Instituto Oswaldo Cruz, Laboratório de Mosquitos Transmissores de Hematozoários, Rio de Janeiro, Brazil; ^3^Centro de Investigación en Salud para el Trópico, Universidad Cooperativa de Colombia, Santa Marta, Colombia; ^4^Instituto Nacional de Salud, Bogotá, Colombia

## Abstract

*Aedes aegypti* is the main vector of the dengue virus in Colombia. Some factors have been associated with its presence; however, in the local context, it has not been sufficiently evaluated. The present study seeks to identify the socioeconomic, environmental, and behavioral factors associated with the presence and abundance of *A. aegypti* in urban dwellings in the municipality of Castilla la Nueva. A cross-sectional cohort study was conducted in houses in the urban area of the municipality of Castilla la Nueva, where 307 houses were sampled by systematic random sampling during May 2018. A multifactorial survey was used to measure the socioeconomic, environmental, and behavioral factors as explanatory variables. The infestation and relative abundance were established by the presence of larval stages and ovitraps. The associated factors for the presence and abundance of *A. aegypti* were identified using negative binomial and logistic regression models. A positive housing infestation of 33.2% was identified by direct inspection and 78.5% with ovitraps. The main factors positively associated with the presence and abundance of *A. aegypti* were one-story homes (PR = 2.26; 95% CI: 1.31–3.87), the storage of water for domestic use (PR = 1.91; 95% CI: 1.18–3.09), and local conditions such as disorganized backyard (PR = 79.95; 95% CI: 10.96–583.24) and the proportion of shade greater than 50% of the backyard (PR = 62.32; 95% CI: 6.47–600.32). And, it is negatively associated with residential gas service (PR = 0.3; 95% CI: 0.16–0.58) and self-administered internal fumigation (PR = 0.37; 95% CI: 0.2–0.69). The presence and abundance of *A. aegypti* were explained by interrelated socioeconomic, environmental, and behavioral factors where local conditions and habits such as the organization of the patio, knowledge about vector biology, and cleaning containers are identified as main topics for future prevention strategies for the transmission of dengue in the local and national context.

## 1. Introduction

*Aedes aegypti* is the primary vector of the dengue fever virus (DFV) in Colombia. Its adaptation to the local anthropic conditions and capabilities of transmission of other viruses such as Zika and chikungunya makes the presence and abundance of this vector a significant threat to public health [1–4]. In 2019, there were 117,339 cases of dengue fever (116,065 classic presentation cases and 1,274 severe presentation cases); the case-fatality proportion of severe dengue was 13.8% (176 deaths) [5]. Additionally, the economic and social impact of the disease has been estimated as 1,198.73 DALYs loss per million, US$225,896,097 costs of medical services and premature deaths, and an estimate of US$104,267,878 in expenses related to epidemiological control and surveillance [6].

So far, prevention strategies for the transmission of DFV have been based on socioeconomic, environmental, and cultural determinants [4, 7, 8]. Several research works have identified social marginality, lack of utilities (drinkable water, residual disposal, and sewage) [9–11], access to health care, land use, and migratory status as the main socioeconomic factors associated with the abundance of the vector [12, 13].

Similarly, other factors such as traditional cultural dynamics, health education, and behavior have been identified as critical [14, 15]. Also, temperature, rainfall, relative humidity, and some specific conditions of the residences are environmental factors that have been associated with the vector presence. In this same dimension, maintenance, state of the patios, and proportion of shadow in the patios have been related to the distribution, abundance, breeding, and mosquito survival [16–19].

The control of vectors in Colombia is conducted following protocols designed for generalized conditions without considering the local particularities. This has hindered the effective execution of programs of vector control and dengue prevention [20–24]. Additionally, the use of the Stegomyia larval index in the regular entomological surveillance programs in Colombia is not adequate to estimate the presence of the vector or the risk of transmission of the vector-borne viral diseases [20, 22]. To overcome these problems, the use of ovitraps and their derived indices have shown higher sensitivity to determine the presence of *A. aegypti* [22, 23, 25, 26]. According to these, there is a research gap on the factors that favor breeding sites and the abundance of the vectors at regional scales.

In Colombia, the lack of utilities such as water supply, sewage, residual disposal, and nonregulated urbanization have been essential factors related to the vector's presence and dengue outbreaks [4]. However, outbreaks of dengue have occurred even in places that do not have such problems, which indicates that other factors are affecting the presence of the vector and the risk of dengue. For example, the municipality of Castilla la Nueva in the Department of Meta has a well-developed urban area with access to basic utilities and has implemented programs to prevent the disease [27]. However, the average incidence of dengue in the last ten years is 2,630 cases per 100,000 person-years (estimated with midyear population), including a peak of 7,436 cases per 100,000 person-years in 2019 [28]. That is why this study aims to identify other socioeconomic, environmental, and behavioral factors associated with the presence and abundance of *A. aegypti* in the municipality of Castilla la Nueva. Our findings allow the identification of parameters that can be used to improve the education, control, and surveillance programs of dengue in local communities.

## 2. Materials and Methods

### 2.1. Study Area

**Figure 1:**
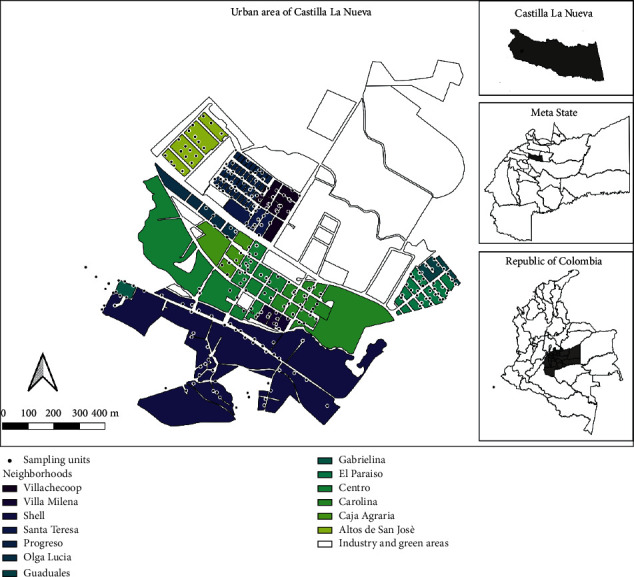
Map of Castilla la Nueva. The map specifies the 13 neighborhoods and each of the survey units and sampling units with ovitraps.

The study was conducted in the urban center of the municipality of Castilla la Nueva, in the Department of Meta, Colombia (3°49′-3°50′N, 73°40′-73°42′W). It has an estimated population of 8,787 (2018 projection), distributed as 4,337(49.4%) in urban areas and 4,450 (50.6%) in rural areas. The urban area has 1008 residential properties in 13 neighborhoods, with approximately 60 blocks (Figure 1). The town of Castilla la Nueva has three types of housing construction: one-story house in regular neighborhoods, an apartment complex (one neighborhood), and a one-floor building with an irregular organization with some precarious housing (nonregulated constructions on public land), which are primarily located closer to the Guamal river. Some of these houses do not have access roads, and they lack at least one utility (domiciliary gas supply) (information available in the geoportal of Instituto Colombiano Agustin Codazzi) [29]. In general, the population of Castilla la Nueva has an excellent social condition, broad coverage in utilities (aqueduct, sewerage, and electricity), as well as in education and health services for its entire population [27].

### 2.2. Study Design and Sampling Strategy

An observational cross-sectional study was designed and conducted. The study was developed during May 2018 in the urban area, taking households as sampling units. The month of May was selected mainly because of the beginning of the rainy season, which is theoretically related to the increase in the abundance of mosquitoes and the beginning of the peaks of dengue cases. The entomological sampling and the delivery of the multifactorial survey were designed systematically in the urban area of Castilla la Nueva. The systematic sampling was carried out according to what was established for the Stegomyia larval index in Colombia [7, 30]; the sample size was calculated using the formula for proportions in a finite set [31]. A 244-house sample was estimated. The sample size was expanded by 30% more, considering the possible losses, empty houses, or the refusal to participate voluntarily in the study, obtaining a total sample size *n* of 317 houses. At the first building in each block, the sampling was conducted, moving clockwise and maintaining a sampling interval every three houses (*N*/*n*), following the national guideline [7]. All the sampled houses were georeferenced. The inclusion criteria for participation were being inhabited, the person responding to the survey should be an adult (18+) and obtain the permission of the owner or tenant. A letter of consent was always presented.

Information bias was controlled with training and standardization of the interviewers, conducting a pilot test, quality control of the survey, and confidentiality of the information, and confounding biases, with binary logistic regression statistical analysis.

A multidimensional survey collected the information; the instrument was prepared in digital format for mobile phones or tablets using the Epi Info application (https://www.cdc.gov/epiinfo/mobile.html). Technicians of the Secretary of Health delivered the survey. The survey was divided into three parts: socioeconomic, environmental, and behavioral.

The following variables were measured in the socioeconomic dimension: age, gender, level of education, income, occupation, type of health insurance, rental or owned, socioeconomic status, number of bedrooms, number of inhabitants of the house, water supply, and garbage collection frequency (Table 1). The variables measured in the environmental dimension were type of residence, concrete washbasins (water tanks), water containers, and building material for walls, floor, and roof. Additionally, maintenance of the house, organization of the patio, and the proportion of shade in the patio were recorded. These conditions are necessary to establish the local conditions index, following the methodology based on Tun-Lin et al. [17] and Manrique-Saide et al. [18] (Table 2). Finally, factors related to knowledge, attitudes, and practices regarding the vector and dengue included the reasoning and motivations for water storage and travel outside the municipality (Table 3).

### 2.3. Entomological Variables

The information regarding the presence of immature stages of *A. aegypti* was generated by direct inspection in potential breeding sites following the methodology established by the Pan American Health Organization and the guidelines for Colombia [7, 30]. Three entomological indicators were used: (1) positive house by the presence/absence of immature stages of *A. aegypti*, (2) positive ovitraps by the presence/absence of positive ovitraps, and (3) the number of eggs per ovitrap. A single sampling was carried out with ovitraps for a month, and eggs collected in the ovitraps were counted using a stereomicroscope at 20 times magnification.

The ovitraps were located inside the houses, mainly in the courtyard, in a shady and cool place, on the floor, or at 1.5 meters high in houses where small children or pets lived. The ovitrap was assembled with a 2-liter plastic bottle cut in half and painted black, and then a strip of white cloth was attached inside. The piece bottle is then filled up with 0.95 liters of water, and 1 gram of *Bacillus thuringiensis* var. *israelensis* (Bti) granulated was added, according to Alarcón et al. [25].

### 2.4. Data Analysis

Exploratory data analysis was conducted to describe the distribution and frequencies of the variables. After this, the association between independent variables and the outcomes was estimated using bivariate cross-tabulation tests (chi-squared test or Fisher's exact test). The prevalence ratio (PR) was calculated for each variable. The adjusted prevalence ratio was calculated using bivariate logistic regression. A multivariate model was built using the variables with significant associations found in the bivariate analysis (*p* value >0.05). Similarly, negative binomial regression models were used to assess the association of variables with the egg counts per ovitrap. The stepwise forward selection was used to select the variables for the final model using the Akaike information criterion (AIC). R statistical software (V: 3.6.1) was used, including the following packages epiDisplay and nortest [32]^.^

### 2.5. Ethical Considerations

Each participant was informed of the aims and implications of study participation. Participation in the study was voluntary, and participants signed a letter of informed consent. The study was authorized by the Institutional Ethical Committee (Comité de Ética Institucional, CES University, Act 116 of 2018) and the department and municipality health branches (Secretaría de Salud del Meta y Secretaría de Salud de Castilla la Nueva).

## 3. Results

### 3.1. Entomological Indices

Three hundred and seven residences (96.8%) from the estimated sample (317 houses) participated in the study (questionnaire and trap allocation). The entomological survey allowed determining a general housing infestation proportion of 33.2% in the urban area of the municipality. The presence of concrete washbasins and other forms of in-house water storage for laundry and other housekeeping tasks contributed the most to the infestation index with 22.48% followed by 200-liter metal barrels (6.84%), pet waterers (1.95%), bottles (0.98%), aquatic plants (0.65%), and flower pots (0.33%). A total of 241 (78.5%) positive residences were identified with the ovitrap sampling method—the number of eggs per ovitrap of *A. Aegypti* was an average of 74.6; 75% of the traps had a capture of fewer than 238 eggs, and the maximum value of eggs was 1.512.

### 3.2. Results of Socioeconomic, Environmental, and Behavioral Dimensions

Most of the participants were women (73.9%). Many participants (76.9%) had at least a high school education, and 1.3% had not received any education. The main occupation identified was housekeeper (44.3%) followed by formal work (22.5%) and self-employed (16.6%). Most of the surveyed population (52.1%) had an income of 2 times the minimum wage in Colombia (approximately US$550). 59.9% of the population owned their house, and all the households in the municipality have access to essential utilities (water, garbage collection, electricity) (Table 1).

76.9% of the residences corresponded to one-story brick and mortar houses, cement roof tiles, tiled floors, and concrete washbasins. 86% of the homes presented good maintenance of painted walls, doors, and windows. However, 24.4% had a disorganized patio, and 35.5% had a partially disorganized patio. In addition, 66.4% of patios had a proportion greater than 50% of shade (Table 2).

As main behavioral factors, it was observed that most of the surveyed population traveled outside the municipality (79.5%), some of them monthly (51.8%). Regarding the habits, it was found that 82.7% of the respondents stated that they wash the reservoirs at least every week. 75.2% of the community stated that the main reason for storing water was domestic activities such as dishwashing, cooking, housekeeping, and laundry.

A significant proportion of the surveyed population demonstrated some knowledge of the vector (94.5%), the breeding sites of the vector (87%), and the role in the transmission of arboviruses (92.2%). However, 39.7% could not describe the behavior and the place of oviposition for *A. aegypti*, which shows that a significant proportion of the population is unaware of important aspects of the biology of the vector.

Regarding the practices to prevent reproduction and mosquito bites, it was found that, in a high percentage of households (77.2%), the people in their homes every eight days generally perform self-application of insecticides. Additionally, they used physical barriers (mosquito nets, window hooks, electric rackets) and controlled actions in the tank, which included washing the tank and some lids that could prevent the entry of mosquitoes (Table 3).

### 3.3. Factors Associated with the Presence of Positive Residences and Positive Ovitraps

The bivariate analysis showed that socioeconomic factors such as the number of bedrooms in residence (between 4 and 6 bedrooms), home ownership, and living in an irregular urban development zone were factors significantly associated with positive houses for the presence of immature *A. aegypti*. In contrast, the factor having domestic gas showed an opposite effect, with a 1.4 times lower probability of finding infested residences than households that do not have this service (PR = 0.51; 95% CI: 0.38–0.69). About the environmental factors, it was found that one-story homes are strongly associated with the presence of vector mosquitoes (PR = 2.26; 95% CI: 1.31–3.87).

The most relevant behavioral factors identified were the storage of water for domestic use (PR = 1.91; 95% CI: 1.18–3.09) and mobility outside the municipality (PR = 0.62; 95% CI: 0.45–0.86), in which the frequency of monthly mobility showed a lower probability of finding infestation with immature mosquito states compared with households where their inhabitants do travel outside the municipality. In addition, it was observed that the people who identified the vector and practice prevention such as regular home pesticide spraying decreased the probability of home infestation 1.85 and 1.6 times, respectively (Table 3).

An association between positive ovitraps and socioeconomic factors such as Internet service and domestic gas was observed since a home with Internet access and gas was less likely to have positive ovitraps. About the environmental factors, it was observed that rustic cement floors (PR = 1.20; 95% CI: 1.05–1.36), houses with moderate maintenance (PR = 1.25; 95% CI: 1.13–1.38), disorganized patios (PR = 1.34; 95% CI: 1.23–1.47), and a proportion of shade greater than 50% in the patio (PR = 1.26; 95% CI: 1.09–1.46) increased the probability of finding positive ovitraps (Tables 1 and 2).

### 3.4. Modeling of Explanatory Factors for the Presence and Abundance of *A. aegypti*

Positive associations with one-story house, undergraduate education level, and owning the home were significant variables associated with the probability of infested residences. On the other hand, having domiciliary gas service and in-house fumigation showed a negative association with the probability of infestation at 3.33 and 2.7 times for larval stages, respectively. Mobility outside the municipality and knowing the vector were also factors identified by the model that decreased the infestation probability; however, these factors were not significant.

The logistic model designed for the positive ovitrap indicator revealed that the main factors associated were a disorganized patio (PR = 79.95; 95% CI: 10.96–583.24) and the proportion of shade greater than 50% of the patio (PR = 62.32; 95% CI: 6.47–600.32). The model also identified that cleaning the concrete washbasins was positively associated with gravid female mosquitoes in the residences (PR = 4.1; 95% CI: 1.64–10.22). The same environmental factors were also positively related to the abundance of *A. aegypti* by the negative binomial regression model for the number of eggs per ovitrap (Table 4).

## 4. Discussion

This study allowed the identification of socioeconomic, environmental, and behavioral factors associated with the presence and abundance of *A. aegypti*. The direct inspection of water containers and the captures of eggs of *A. aegypti* with ovitraps were adequate for the identification of the presence of the vectors as it has been reported by other studies [18, 23, 33–37]. Likewise, egg counts in ovitraps have shown to effectively determine the relative presence and abundance in a specific location since these counts have been correlated to the number of adults and the risk of viral transmission [24, 25, 38, 39].

The percentage of residences with the presence of the mosquito was 32.2%, which is similar to that reported by other studies [21, 40, 41]. However, this study shows that 200 L containers as the main breeding sites for *A. aegypti* (concrete washbasins, low tanks, laundry tanks, and barrel containers). These containers have been identified as the most prolific breeding sites (>60%) for mosquito production in Colombia [21, 40, 42, 43]. The ovitraps showed a better sensitivity to identify female mosquito intrusion than direct inspection of water containers finding a proportion of positivity twice the house index. This result is similar to that by Alarcon et al. [25] in the municipalities of Apartadó and Carepa, where the proportion of ovitraps was higher than 70% for both municipalities, while the proportion of positive residences varied between 1.92% and 58.2%. Other studies have also reported similar results in other municipalities and other countries such as Thailand and Mexico, suggesting that ovitraps are sensitive enough to determine the presence of the vector and can be useful as a tool for entomological surveillance [23, 26].

In the present study, the socioeconomic variables associated with the vector's presence were the number of bedrooms in the house, property condition (rent/own), and some services such as gas and Internet. However, the latter two variables are not similar to those used in other studies [12, 34]. A study in Ecuador showed that many families in the same house with owners older than 35 years were identified as risk factors of vector presence [36]. These findings are comparable to our study where houses with many rooms, habited by owners older than 39 years, were associated with mosquito vectors. To the extent of our knowledge, there are no reports of domestic gas and Internet service associated with the presence of the vector, so this would be the first report of this association. Association with other utilities such as drinkable water, sewage, and residual disposal has been reported previously [9, 10, 36, 37]. Internet access and access to basic utilities are related to access to knowledge and better living conditions related to positive preventive behavior [44].

Other research works in Colombia (Melgar, Girardot, and Villavicencio) have shown that houses in privileged neighborhoods have less density of the vector, thus less risk of exposure to dengue [12, 45]. However, in this study, the proportion of positive residences did not differ among the different socioeconomic levels as evidence that neither the type of construction nor the urban condition in the neighborhoods affects the presence of the vector. In this case, we believe that the presence of *A. aegypti* in the study areas is related to the environmental conditions rather than the socioeconomic level.

Regarding environmental factors, temperature has been identified as an important factor in the abundance, dispersion, and transmission of arboviruses by the mosquito [46–48]. However, in the tropics, the temperature does not have drastic variations during the year; other factors such as the living spaces and neighborhood characteristics become more important. In this study, we found that houses with concrete floors, poor maintenance, poor organization, and with a proportion of shade in the patio higher than 25% are houses with a higher probability of finding immature stages of mosquitoes. These results are similar to those reported in studies conducted in Bangladesh and Ecuador where the partial shading of the patio and inadequate maintenance of the house and patio increased the probability of finding the vector 13 times [35, 36].

Other factors related to the presence of the vector were behavioral factors. Habits and monthly frequency of travel out of the municipality showed a low probability of finding the mosquito in the study area. This suggests that people who travel with less frequency could permanently control breeding sites, minimizing the mosquito reproduction. These results are similar to those reported in studies of dengue transmission models by Stoddard et al. [49], where they determined that the basic reproductive number (R0) was 1.3 for exposure of the vector in house, compared with 3.75 when the exposure occurred elsewhere (e.g., schools or markets). Even though this model does not explain the vector-factor relationship, it shows a comparative framework of the potential importance of the mobility of people at risk and the relation of these with the vector and the capability of virus transmission.

The reasons and motivations for storing water in houses' containers are behavioral factors to consider. In this municipality, all the population has access to tap water with constant supply; however, people decide to store water in the houses for, according to them, its use in “multiple housekeeping chores.” The presence of water containers was associated with a higher probability of the presence of the mosquito. This same association was found in a study in Dhaka, Bangladesh, where they found a threefold risk of the presence of the vector if water was stored compared with those residences where water was not stored [34]. Our results suggest the need to implement programs focused on modifying the practice of water storage without altering the activities in the houses.

We also evaluated the potential association of knowledge about the vector and dengue with the presence of the vector. Widespread global recommendations of control and prevention of mosquito populations and dengue transmission always include knowledge access and appropriation as essential [8]. A study in Ecuador evaluated vector knowledge before, during, and after the rainy season and found that during the rainy season, an education-based intervention had a preventive effect on the presence of the vector [36]. In the present study, a similar effect between knowledge and the presence of the vector was identified, which suggests expanding educational processes in different weather seasons as preventive strategies.

On the other hand, we found an association between spraying insecticide (by the inhabitants) and the presence of the vector. This effect was also reported in a systematic review by Samuel et al. [50], where insecticide sprays reduce the abundance of larval and adult forms of the mosquito and decrease in 4.3 times the risk of dengue fever. Indicating for our study that habits related to the self-application of insecticides and having an effect on mosquitoes is an acquired behavior, focusing on other physical or biological control methods with a lower risk of failure (resistance to insecticides).

The logistic model identified multiple explanatory factors associated with the presence of the vector in the participant households. The main associated factors were the house type, being an owner, and having professional habitants. These findings were surprising since they are characterized by the quality of life and possibly by a better understanding and attitudes toward preventive behaviors for controlling the vector. However, our results were similar to those reported in other studies in Cali [51], Java [52], and Thailand [53], where they demonstrated that people living in higher-income neighborhoods, have higher education levels, and have better jobs have higher risks of getting dengue fever. Regarding the type of residence, a traditional residence type older than 20 years of construction has been found to be associated with the presence of the mosquito [54].

Explanatory models developed with positive ovitraps and the number of eggs per ovitrap found environmental factors associated with the presence of gravid females. The local conditions as poor maintenance, >50% patio shadow, and disorganized patio were associated with the presence and abundance of *A. aegypti*. These factors can also be related to the educational level, the quality of life, and other socioeconomic factors that promote the conditions for reproduction and the presence of the vector [55]. These factors have been reported previously in association with the presence of the vector and as for the risk of dengue transmission [18, 56].

The model also identified that washing the concrete washbasins is associated with the presence and abundance of the vector. However, the information of practices obtained may be influenced by participants' answers since this practice is perceived as an accepted norm behavior. Also, we found that participants reporting washing the concrete washbasins weekly in participants' residences reported a higher infestation and abundance of the vector. These findings show that people know the importance of concrete washbasins' cleanliness and frequency of cleaning, but this knowledge does not affect the behavior or the cleaning is not being performed adequately. The results are consistent with those reported in a study conducted in Bucaramanga, where an intervention of information, education, and communication was implemented. This intervention showed a modification of the knowledge about vector control practices, but this did not decrease the number of breeding sites or the incidence of dengue [57].

One limitation of this study is that we determined the presence and abundance of the immature stages of the vector (larvae, pupa, and eggs) without using the Stegomyia larval index or density or abundance of pupas or adults, important outcome indexes that might have shown an association with other factors. Second, the study's cross-sectional nature only allowed collecting data at a specific time of the year (rain season). We consider that collecting information in the dry season could have led to different results since the vector's abundance and presence could change in these two seasons.

## 5. Conclusion

The present study identified that the presence and abundance of *A. aegypti* in the study area are explained by socioeconomic, environmental, and behavioral factors such as home ownership status, housing type (traditional house older than 20 years of construction), service domestic gas, spray the house with insecticides, education level, profession, patio condition, shadow in the patio, and concrete washbasins cleaning, where the conditions of the residences and modes of living of its inhabitants played an important role. Additionally, the study concluded that the main mosquito breeding sites are large water containers larger than 200 liters in which entomological and community control actions should be focused. Likewise, the use of ovitraps as an entomological indicator of the presence of *A. aegypti* was shown to be more sensitive than direct inspection of water containers, suggesting that this tool could be used routinely for entomological surveillance in Colombia.

Finally, based on the identified factors, we recommend generating public policies focused on house maintenance, strategies that highlight the importance of patio organization, and adequate water container cleaning as topics in preventive education programs. In addition to these findings, efforts should be made to help the people to go from knowledge to practice. We believe that the implementation of these activities will help decrease the presence and abundance of the vector, thus reducing the risk of exposure to the dengue fever virus.

## Figures and Tables

**Table 1:** Frequencies (*n*, %) of socioeconomic factors associated with identifying positive houses for immature stages of *A. aegypti* (PR, 95% CI; *χ*^2^) and factors associated with the presence of positive ovitraps (PR, 95% CI; *χ*^2^).

Variables	*n* (%)	Positive houses				Positive ovitraps			
		Yes	No	PR (95% CI)	*χ* ^2^	Yes	No	PR (95% CI)	*χ* ^2^
Sex									
Female	227 (73.9)	76	151	1.03 (0.72, 1.48)	0.0005	177	50	0.97 (0.86, 1.11)	0.049
Male	80 (26.1)	26	54	1.00		64	16	1.00	

*Age*									
From 18 to 39	144 (46.9)	99	45	1.00	1.51	32	112	1.00	0.88
From 40 to 60	130 (42.3)	87	43	1 (0.86, 1.18)		29	101	0.99 (0.56, 1.77)	
>60	33 (10.7)	19	14	0.85 (0.63, 1.15)		5	28	1.6 (0.57, 4.48)	

*Education level*									
None	4 (1.3)	3	1	12 (0.51, 280.09)	10.0	3	1	0.95 (0.54, 1.69)	6.21
Primary	97 (31.6)	31	66	1.88 (0.2, 17.52)		77	20	1.02 (0.90, 1.15)	
High school	139 (45.3)	44	95	1.85 (0.2, 17.06)		111	28	1.03 (0.92, 1.16)	
Technician or technologist	52 (16.9)	16	36	1.78 (0.18, 17.19)		42	10	1.03 (0.89, 1.20)	
Professional	10 (3.3)	7	3	9.33 (0.71, 122.57)		5	5	0.63 (0.34, 1.17)	
Postgraduate	5 (1.6)	1	4	1.00		3	2	1.00	

*Occupation*									
Housewife	136 (44.3)	38	98	1.00	8.19	103	33	1.00	3.53
Unemployed	32 (10.4)	11	21	1.35 (0.59, 3.07)		27	5	1.08 (0.92, 1.27)	
Employee	69 (22.5)	26	43	1.56 (0.84, 2.88)		55	14	1.02 (0.89, 1.17)	
Student	15 (4.9)	5	10	1.29 (0.41, 4.02)		11	4	0.93 (0.68, 1.27)	
Independent worker	51 (16.6)	20	31	1.66 (0.85, 3.27)		42	9	1.06 (0.92, 1.22)	
None	2 (0.7)	2	0	1.E+07 (0, Inf)		1	1	0.64 (0.16, 2.54)	
Pensioner	2 (0.7)	0	2	0 (0, Inf)		2	0	2.E+06 (0, Inf)	

*Family income*									
≤1 MW	147 (47.9)	51	96	1.09 (0.79, 1.49)	3.76	120	27	1.08 (0.96, 1.21)	5.2
1-2 MW	145 (47.2)	43	102	0.81 (0.59, 1.12)		108	37	0.91 (0.81, 1.02)	
3-4 MW	9 (2.9)	5	4	1.71 (0.93, 3.13)		9	0	8.*E* + 06 (0, Inf)	
>4 MW	6 (2)	3	3	1.00		4	2	1.00	

*Number of people*									
From 1 to 5	261 (85)	175	86	1.00	1.53	55	206	1.00	m
From 6 to 9	43 (14)	29	14	0.98 (0.49, 1.95)		9	34	1.01 (0.46, 2.23)	
>10	3 (1)	1	2	4.07 (0.36, 45.51)		2	1	0.13 (0.01, 1.5)	

*Number of rooms*									
From 1 to 3	262 (85.3)	182	80	1.00	**7.95** ^*∗*^	58	204	1.00	0.45
From 4 to 6	40 (13)	19	21	**2.51 (1.28, 4.93)**		7	33	1.34 (0.56, 3.19)	
> 7	5 (1.6)	4	1	0.57 (0.06, 5.17)		1	4	1.14 (0.12, 10.37)	

*Overcrowding*									
From 0.2 to 1.8	217 (70.7)	140	77	1.00	1.71	42	175	1.00	2.96
From 2 to 2.7	69 (22.5)	50	19	0.69 (0.38, 1.26)		20	49	1.5 (0.96, 2.36)	
> 2.9	21 (6.8)	15	6	0.73 (0.27, 1.95)		4	17	0.88 (0.35, 2.18)	

*Property condition*									
Own	184 (59.9)	71	113	**1.26 (1.06, 1.51)**	**5.36** ^*∗*^	147	37	1.05 (0.93, 1.18)	0.34
Rent	123 (40.1)	31	92	1.00		94	29	1.00	

*Water supply service*									
Yes	307 (100)	102	205	NA	NA	241	66	NA	NA

*Water supply frequency*									
Every day	307 (100)	102	205	NA	NA	241	66	NA	NA

*Garbage collection service*									
Yes	307 (100)	102	205	NA	NA	241	66	NA	NA

*Garbage collection frequency*									
From 2 to 3 days	307 (100)	102	205	NA	NA	241	66	NA	NA

*Electric service*									
Yes	307 (100)	102	205	NA	NA	241	66	NA	NA

*Cable television service*									
Yes	250 (81.4)	79	171	0.78 (0.54, 1.13)	1.23	192	58	0.89 (0.79, 1.01)	1.80
No	57 (18.6)	23	34	1.00		49	8	1.00	

*Internet service*									
Yes	159 (51.8)	49	110	0.86 (0.63, 1.18)	0.65	116	43	**0.86 (0.77, 0.97)**	**5.35** ^*∗*^
No	148 (48.2)	53	95	1.00		125	23	1.00	

*Service domestic gas*									
Yes	251 (81.8)	71	180	**0.51 (0.38, 0.69)**	**13.9** ^*∗*^	191	60	**0.85 (0.76, 0.96)**	**3.97** ^*∗*^
No	56 (18.2)	31	25	1.00		50	6	1.00	

*Socioeconomic levels*									
Level 1	113 (36.8)	39	74	0.94 (0.5, 1.78)	0.65	87	26	0.97 (0.86, 1.10)	0.69
Level 2	127 (41.4)	39	88	0.79 (0.42, 1.48)		99	28	0.99 (0.88, 1.11)	
Level 3	67 (21.8)	24	43	1.00		55	12	1.00	

*Irregular urbanization area*									
Si	42 (86.3)	20	22	**1.82 (1.05, 3.18)**	**4.54** ^*∗*^	36	6	1.64 (0.72, 3.73)	1.49
No	265 (13.7)	82	183	1.00		205	60	1.00	

*Health regime*									
Subsidized	184 (59.9)	61	123	1.01 (0.73, 1.39)	1.03*e* − 30	147	37	0.96 (0.85, 1.08)	0.34
Contributory	123 (40.1)	41	82	1.00		94	29	1.00	

MW = minimum wage in Colombia. Bold number and asterisk represent statistical significance of *p* < 0.05.

**Table 2:** Frequencies (*n*, %) of environmental factors associated with identifying positive houses with immature stages of *A. aegypti* (PR, 95% CI; *χ*^2^) and in the presence of positive ovitraps (PR, 95% CI; *χ*^2^).

Variables	*n* (%)	Positive houses				Positive ovitraps			
		Yes	No	PR (95% CI)	*χ* ^2^	Yes	No	PR (95% CI)	*χ* ^2^
*Housing type*									
House	236 (76.9)	90	146	**2.26 (1.31, 3.87)**	**10.2** ^*∗*^	189	47	1.09 (0.94, 1.28)	1.14
Apartment	71 (23.1)	12	59	1.00		52	19	1.00	

*Wall type*									
Cement	289 (94.1)	97	192	1.00	2.04	226	63	1.00	5.63
Plastic fiber	1 (0.3)	0	1	0		1	0	1.28 (1.20, 1.35)	
Wood	14 (4.6)	5	9	1.08 (0.52, 2.22)		13	1	1.19 (1.02, 1.40)	
Zinc sheet	3 (1)	0	3	0		1	2	0.42 (0.09, 2.09)	

*Floor type*									
Tile	276 (89.9)	93	183	1.00	2.54	215	61	1.00	**7.39** ^*∗*^
Cement	26 (8.5)	9	17	1.05 (0.60, 1.82)		24	2	**1.18 (1.04, 1.34)**	
Land	5 (1.6)	0	5	0		2	3	0.51 (0.17, 1.48)	

*Roof type*									
Eternit roof tile	289 (94.1)	97	192	1.2 (0.56, 2.59)	0.06	226	63	0.94 (0.76, 1.16)	0.05
Zinc tile	18 (5.9)	5	13	1.00		15	3	1.00	

*Concrete washbasins (water tanks or low tank)*									
Yes	269 (87.6)	95	174	1.92 (0.96, 3.82)	3.56	215	54	1.17 (0.93, 1.46)	1.97
No	38 (12.4)	7	31	1.00		26	12	1.00	

*Other types of water container*									
Yes	113 (36.8)	33	80	0.82 (0.58, 1.16)	1.03	85	28	0.94 (0.82, 1.06)	0.85
No	194 (63.2)	69	125	1.00		156	38	1.00	

*More than one water container*									
Yes	75 (24.4)	26	49	1.06 (0.70, 1.61)	0.09	59	16	1.01 (0.62, 1.63)	0.001
No	232 (75.6)	76	156	1.00		182	50	1.00	

*House maintenance*									
Well maintained	264 (86)	84	180	1.00	2.70	200	64	1.00	**8.43** ^*∗*^
Moderately maintained	25 (8.1)	12	13	1.50 (0.97, 2.34)		24	1	**1.25 (1.13, 1.38)**	
Poor maintained	18 (5.9)	6	12	1.00 (0.51, 1.97)		17	1	**1.22 (1.07, 1.39)**	

*Patio (backyard) condition*									
Tidy	123 (40.1)	39	84	1.00	0.22	63	60	1.00	**90.5** ^*∗*^
Moderately tidy	109 (35.5)	37	72	1.03 (0.74, 1.44)		105	4	**1.40 (1.27, 1.55)**	
Untidy	75 (24.4)	26	49	1.06 (0.74, 1.52)		73	2	**1.34 (1.23, 1.47)**	

*Shadow in the patio*									
<25%	20 (6.5)	9	11	1.00	1.68	1	19	1.00	**68.7** ^*∗*^
25–50%	83 (27)	29	54	1.07 (0.76, 1.52)		68	15	1.06 (0.94, 1.20)	
>50%	204 (66.4)	64	140	0.85 (0.62, 1.18)		172	32	**1.26 (1.09, 1.46)**	

Bold number and asterisk represent statistical significance of *p* < 0.05.

**Table 3:** Frequencies (*n*, %) of behavioral factors associated with identifying positive houses with immature stages of *A. aegypti* (PR, 95% CI; *χ*^2^) and factors associated with the presence of positive ovitraps (PR, 95% CI; *χ*^2^).

Variables	*n* (%)	Positive houses				Positive ovitraps			
		Yes	No	PR (95% CI)	*χ* ^2^	Yes	No	PR (95% CI)	*χ* ^2^
*Mobility outside the municipality*									
Yes	244 (79.5)	72	172	**0.62 (0.45, 0.86)**	**6.61** ^*∗*^	189	55	0.94 (0.82, 1.08)	0.49
No	63 (20.5)	30	33	1.00		52	11	1.00	

*Frequency of mobility outside the municipality*									
Does not travel	61 (19.9)	29	32	1.00	**9.66** ^*∗*^	50	11	1.00	6.03
Daily	8 (2.6)	3	5	0.79 (0.31, 2.00)		4	4	0.63 (0.31, 1.26)	
Weekly	35 (11.4)	11	24	0.66 (0.38, 1.15)		29	6	1.06 (0.90, 1.25)	
Every 15 days	44 (14.3)	17	27	0.81 (0.51, 1.28)		37	7	1.08 (0.94, 1.25)	
Monthly	159 (51.8)	42	117	**0.55 (0.38, 0.80)**		121	38	0.94 (0.84, 1.05)	

*Concrete washbasins cleaning*									
Yes	254 (82.7)	84	170	0.97 (0.64, 1.47)	1.91*e* − 30	205	49	1.19 (0.98, 1.44)	3.52
No	53 (17.3)	18	35	1.00		36	17	1.00	

*Frequency of concrete washbasins cleaning*									
It has no tank	38 (12.4)	7	31	1.00	6.68	26	12	1.00	5.39
From 2 to 8 days	249 (81.1)	85	164	1.16 (0.75, 1.80)		200	49	1.88 (0.89, 4)	
From 9 to 15 days	15 (4.9)	7	8	1.43 (0.81, 2.53)		10	5	0.92 (0.26, 3.3)	
From 20 to 30 days	5 (1.6)	3	2	1.83 (0.88, 3.81)		5	0	3.E+04 (0, Inf)	

*Identify covered water containers*									
Yes	38 (12.4)	15	23	1.00	0.48	33	5	1.00	1.27
No	269 (87.6)	87	182	1.22 (0.79, 1.88)		208	61	0.89 (0.77, 1.02)	

*Reasons for water storage*									
Custom	29 (9.4)	7	22	0.71 (0.36, 1.37)	**8.69** ^*∗*^	23	6	1.01 (0.83, 1.23)	1.25
Does not store water	47 (15.3)	8	39	1.00		34	13	1.00	
Domestic use	231 (75.2)	87	144	**1.91 (1.18, 3.09)**		184	47	1.06 (0.92, 1.23)	

*Knowledge of the biological behavior of the vector*									
Yes	185 (60.3)	58	127	1.00	0.54	147	38	1.00	0.13
No	122 (39.7)	44	78	1.15 (0.74, 1.77)		94	28	0.97 (0.86, 1.09)	

*Knowledge of breeding sites*									
Yes	267 (87)	87	180	1.00	0.19	209	58	1.00	2.*E* − 03
No	40 (13)	15	25	1.85 (1.2, 2.8)		32	8	1.02 (0.86, 1.2)	

*Vector knowledge*									
Yes	290 (94.5)	92	198	**0.54 (0.35, 0.83)**	**4.16** ^*∗*^	229	61	1.00	0.26
No	17 (5.5)	10	7	1.00		12	5	0.89 (0.65, 1.22)	

*Knowledge of arboviruses*									
Yes	283 (92.2)	90	193	1.00	2.53	222	61	1.00	5.3*E* − 30
No	24 (7.8)	12	12	1.57 (1.02, 2.43)		19	5	1.00 (0.81, 1.25)	

*Biting control strategy*									
Physical	229 (74.6)	78	151	1.11 (0.76, 1.62)	0.15	184	45	1.10 (0.95, 1.28)	1.42
Chemistry	78 (25.4)	24	54	1		57	21	1	

*Breeding site control strategies*									
Actions on the tank	234 (76.2)	81	153	1.20 (0.81, 1.80)	0.61	182	52	0.96 (0.84, 1.10)	0.15
Use of insecticides and chlorine	73 (23.8)	21	52	1		59	14	1	

*Spray the house with insecticides*									
Yes	237 (77.2)	69	168	1.00	**7.12** ^*∗*^	189	48	1.00	0.66
No	70 (22.8)	33	37	**1.61 (1.17, 2.22)**		52	18	0.93 (0.80, 1.00)	

*Fumigation frequency*									
From 1 to 8 days	261 (85)	169	92	1.00	4.61	54	207	1.00	0.69
Every 15 days	11 (3.6)	7	4	0.95 (0.60, 1.49)		3	8	0.92 (0.64, 1,33)	
Monthly	35 (11.4)	29	6	1.28 (1.07, 1.59)		9	26	0.94 (0.77, 1.15)	

*How do you spray insecticides?*									
Self-application	229 (74.6)	67	162	**0.65 (0.47, 0.90)**	**7.98** ^*∗*^	183	46	1.07 (0.92, 1.25)	1.07
Contract fumigation	8 (2.6)	2	6	0.75 (0.22, 2.51)		6	2	1.00 (0.66, 1.54)	
Do not spray	70 (22.8)	33	37	1.00		52	18	1.00	

Bold number and asterisk represent statistical significance of *p* < 0.05.

**Table 4:** Explanatory models for the entomological indicators positive house, positive ovitrap, and the number of eggs per ovitrap in Castilla la Nueva.

Parameter	PR (95% CI)	Adj. PR (95% CI)	*p* value
*Explanatory model for houses positive to larval stages (logistic model)*			
Housing type: house	3.03 (1.54, 5.95)	3.38 (1.64, 6.99)	<0.001
Mobility outside the municipality: yes	0.46 (0.26, 0.81)	0.55 (0.29, 1.03)	0.065
Vector knowledge: yes	0.33 (0.12, 0.88)	0.4 (0.13, 1.25)	0.114
Service domestic gas: yes	0.32 (0.18, 0.58)	0.3 (0.16, 0.58)	<0.001
Spray the house with insecticides: yes	0.46 (0.27, 0.8)	0.37 (0.2, 0.69)	0.002
Education level: professional	4.96 (1.26, 19.61)	7.53 (1.59, 35.72)	0.007
Property condition: own	1.86 (1.13, 3.09)	1.96 (1.12, 3.44)	0.016

*Explanatory model for houses with positive ovitraps (logistic model)*			
Patio condition: Moderately tidy	11.97 (4.22, 33.95)	27.42 (7.78, 96.62)	<0.001
Patio condition: untidy	13.9 (3.31, 58.33)	79.95 (10.96, 583.24)	<0.001
Shadow in the patio: 25–50%	1.34 (0.7, 2.54)	57.62 (5.53, 599.82)	<0.001
Shadow in the patio: >50%	2.65 (1.52, 4.63)	62.32 (6.47, 600.32)	<0.001
Concrete washbasins cleaning: yes	1.98 (1.03, 3.81)	4.1 (1.64, 10.22)	0.002

*Explanatory model for abundance by number of eggs (negative binomial model)*			
Patio condition: moderately tidy	3.32 (2.25, 4.91)	—	<0.001
Patio condition: untidy	6.77 (4.40, 10.63)	—	<0.001
Shadow in the patio: 25–50%	56.35 (23.53, 125.27)	—	<0.001
Shadow in the patio: >50%	84.67 (37.02, 176.66)	—	<0.001

## Data Availability

All relevant study data are within the manuscript.

## Abbreviations

Ae: Aedes
DFV: Dengue fever virus
Bti: *Bacillus thuringiensis* var. *israelensis*
PR: Prevalence ratio
AIC: Akaike information criterion.
